# The Unspoken Wounds: Understanding the Psychological Impact on Healthcare Professionals Fighting COVID-19 in Pakistan

**DOI:** 10.1155/2024/3364960

**Published:** 2024-02-29

**Authors:** Rimsha Khan, Hasnain Javed, Warda Fatima, Ali Ahsan, Malik Ihsan Ullah Khan, Saeed Ahmad, Mohsin Khurshid

**Affiliations:** ^1^Provincial Public Health Reference Laboratory, Punjab AIDS Control Program, Primary and Secondary Healthcare Department, Lahore, Pakistan; ^2^Institute of Biochemistry and Biotechnology, University of Veterinary and Animal Sciences Lahore, Lahore, Pakistan; ^3^Institute of Microbiology and Molecular Genetics, University of the Punjab, Lahore, Pakistan; ^4^University Institute of Medical Laboratory Technology, Faculty of Allied Health Sciences, The University of Lahore, Lahore, Pakistan; ^5^Institute of Molecular Biology and Biotechnology, The University of Lahore, Lahore, Pakistan; ^6^Centres for Disease Control and Prevention (CDC-US), Islamabad, Pakistan; ^7^Institute of Microbiology, Government College University Faisalabad, Faisalabad, Pakistan

## Abstract

During the COVID-19 pandemic, hospital staff faced numerous mental health challenges. However, limited research focused on anxiety and stress specifically among hospital workers during this time. Therefore, this study aimed to investigate the anxiety levels of healthcare workers during the COVID-19 pandemic. A multidimensional, cross-sectional survey was distributed to healthcare workers and staff at hospitals, COVID-19 laboratories, and healthcare settings. The survey included a total of 625 frontline healthcare workers, with 445 (71.2%) being male and 180 (28.8%) female. There were 405 (64.8%) lab professionals, 90 (14.0%) doctors, and 130 (20.8%) others, including nursing staff, administrative personnel, and supporting staff crucial to the functioning of healthcare settings. Among the lab professionals, 37.0% reported moderate depression levels and 16.0% reported severe depression levels during the pandemic. For doctors, 22.2% experienced mild depression and 33.33% experienced severe depression. Several factors were significantly associated with depression and anxiety among frontline healthcare workers, including physiological and social factors, fear of infection, risk of infecting family members and colleagues, lack of personal protective equipment (PPE), long working hours, untrained staff, social issues, and cooperation problems. These factors collectively contributed to reduced work efficacy during the pandemic. Frontline health workers played a critical role in the fight against COVID-19. The findings from this study have important implications for developing strategies to improve the mental health of healthcare workers during the pandemic and implementing policies that enhance work efficacy, ultimately leading to the improved outcomes.

## 1. Introduction

The emergence of SARS-CoV-2, the virus responsible for COVID-19 pandemic, has severely strained healthcare systems worldwide, presenting unique challenges in various aspects of life and medicine [1]. The COVID-19 pandemic has caused a significant public health crisis [2], and placed immense pressure on healthcare workers and services. The surge in COVID-19 cases has strained healthcare resources, including medications, ICU beds, ventilators, and personal protective equipment (PPE). As a result, healthcare workers on the frontlines of the pandemic have experienced high levels of stress and anxiety [3, 4]. These frontline workers are at a significant risk of developing mental health issues, as observed during previous outbreaks like Ebola and SARS [5–7]. Doctors and healthcare professionals involved in diagnosing and treating COVID-19 patients are particularly vulnerable to mental health problems and stress [5, 8]. Access to the healthcare services, including timely and effective treatment, is a crucial aspect that can influence the mental well-being of healthcare workers. Several factors contribute to the emotional strain experienced by healthcare workers, including the growing number of cases, limited resources for patient care, heavy workloads, shortages of medications and PPE, media pressure, and inadequate support [8, 9]. Studies have shown that many healthcare workers fear infecting their families and friends [10]. They feel insecure, ashamed, and uncomfortable in their work, often experiencing depression, anxiety, and stress, and even contemplating resignation [11, 12].

Public health emergencies have a significant impact on the health, safety, and well-being of individuals and populations, often resulting in emotional responses such as psychological problems and depression [13, 14]. Studies have shown that during the SARS epidemic, approximately 29%–35% of healthcare workers experienced mental stress [15]. Furthermore, even after several years had passed since the outbreak, it was observed that 10% of the healthcare staff reported symptoms of posttraumatic stress disorder (PTSD) [16]. Specifically, those who worked in wards treating infected patients were found to be at a higher risk of developing PTSD [17]. Previous research has concluded that healthcare workers, particularly those on the frontline of the COVID-19 pandemic such as doctors, nurses, and laboratory testing staff, are at a heightened risk of developing mental health issues and may require psychological interventions. Moreover, individuals with preexisting mental illnesses are also at a higher risk of developing further mental health problems when combined with these additional risk factors. These risk factors can lead to a range of issues, including insomnia, anxiety disorders, stress, depression, extreme fear of illness, social isolation, anger, changes in behavior, mood swings, posttraumatic stress disorders, somatization, physical health disorders, and fear-related disorders [18–20].

In Pakistan, more than 5,000 healthcare workers were diagnosed with COVID-19, and unfortunately, 58 healthcare workers lost their lives to the virus [21]. These individuals, representing a spectrum of professions within the healthcare sector, devoted their lives to the well-being of others. From frontline nurses battling the virus to dedicated support staff managing critical logistics, each worker played a vital role in the complex healthcare ecosystem. We honor their memory, emphasizing their contributions and the unique challenges faced by healthcare professionals on the frontlines of the pandemic.

The primary objective of this study is to assess the prevalence of depression and anxiety symptoms among various healthcare workers, such as physicians, pharmacists, nurses, and laboratory workers, who have been directly affected by the COVID-19 outbreak in Pakistan. Furthermore, the study aims to investigate the factors that influence the mental health of healthcare workers, specifically focusing on identifying subgroups that are more vulnerable to experiencing psychological effects during the COVID-19 pandemic. The findings from this study will be valuable in identifying effective protective strategies that can be implemented to prioritize the mental well-being of healthcare workers. Additionally, the study will contribute to the development of targeted interventions for mental health care, ensuring that systematic and organizational support is provided to enhance the overall well-being of healthcare workers.

## 2. Materials and Methods

### 2.1. Ethics Approval and Study Design

This study received ethics approval from the Academic and Research Unit of the Provincial Public Health Reference Laboratory, Punjab AIDS Control Program, Primary and Secondary Healthcare Department in Lahore, Pakistan (Ref: PACP/15-A/22). A cross-sectional survey was designed during the peak of the pandemic to gather information from healthcare workers on the frontline of COVID-19, including doctors, nurses, physicians, pharmacists, and laboratory staff, in Pakistan. The workload and burden associated with COVID-19 were particularly high in the Punjab Province, which was chosen as the study site. The workload and burden associated with COVID-19 was particularly high in the Punjab Province due to high-population density, well-managed healthcare infrastructure, which has made Punjab a focal point of the pandemic's impact. However, participants from other regions also took part in the study because this survey was disseminated through various social media platforms, including WhatsApp, Facebook, LinkedIn, and Instagram, to reach healthcare workers in different regions of the country. The survey was conducted over a period of 3 months, coinciding with the third wave of the COVID-19 pandemic in Pakistan. To adhere to the government's instructions to stay at home and prevent the spread of COVID-19, the survey was conducted online, as it proved challenging and impractical to conduct it offline given the circumstances and consequences of the pandemic.

### 2.2. Survey Tool

A comprehensive literature review was conducted to inform the design of the survey for this study [7, 13, 17, 22, 23]. To validate the questionnaire, a committee consisting of senior health professionals, provincial laboratory incharge, medical doctors, nurses, and medical laboratory technologists was consulted. The questionnaire comprised of yes/no and multiple-choice questions. Prior to initiating the E-questionnaire, participants provided their consent. The questionnaire consisted of three sections.

The first section collected participants' demographic information, including their healthcare worker type, gender, age, marital status, place of residence, and education. The second section contained questions pertaining to COVID-19 exposure, patient health inquiries, and anxiety disorders. The third section aimed to assess the mental health of the participants. This type of study questionnaire has been widely used and validated in various populations as a screening measure for depression and anxiety [23–25]. Furthermore, these tools have also been validated for the Pakistani population in previous studies [26, 27].

In the last section, participants were asked about whether they had sought any psychological services to address their mental health issues. Additionally, they were asked to report their overall health status as “very poor” to “very good.” The questionnaire also included inquiries about demographics, mental stress experienced during the pandemic, recognition from the government in terms of allowances and salary increases, family and societal pressures, free vaccination, and free diagnostic tests.

### 2.3. Sampling and Sample Size

To establish the appropriate sample size for this study, the guidelines provided by the World Health Organization (WHO) were followed [28, 29]. The study specifically targeted healthcare professionals, including workers, doctors, nurses, laboratory workers, and lab technicians. A total of 625 responses were obtained for the survey. The questionnaire incorporated a mix of open-ended, close-ended, and Likert-scale-based questions. All participants were required to be above 20 years of age and possess a minimum of 16 years of education. To ensure comprehensibility, the questions were presented in both English and Urdu (the national language of Pakistan).

### 2.4. Data Collection

The data collection for this study utilized the snowball sampling technique, leveraging various social media platforms. These platforms included WhatsApp, Facebook, LinkedIn, and Instagram. The survey was shared with participants, who were then requested to share it with other healthcare workers within their network. The survey was easily accessible through a provided link, and the aims of the study were clearly stated on the first page. Additionally, the survey included information about confidentiality, consent, the right to withdraw, and voluntary participation. Participants were required to provide informed consent before participating in the study. The survey encompassed both frontline and nonfrontline healthcare workers residing in Pakistan.

### 2.5. Data Management and Analysis

The collected data were subjected to the statistical analysis using SPSS version 20 (IBM Corp., Armonk, NY, USA) [20]. Statistical differences between different groups were assessed using *p*-values. Percentages, central tendencies, and charts were calculated using Microsoft Excel 13. Descriptive analysis of the data was conducted using SPSS 17. Outliers in the data were identified and analyzed using the Spearman's test for heteroscedasticity using two-way ANOVA on GraphPad Prism 6.04.

## 3. Results

### 3.1. Sociodemographic Characteristics of the Participants

A total of 625 frontline healthcare professionals willingly participated in this survey by providing their consent. Among these participants, approximately one-third were male, accounting for about 445 individuals (71.2%), while the remaining 180 individuals (28.8%) were female. According to Table 1, it was found that female respondents experienced higher levels of depression compared to their male counterparts.

Regarding marital status, slightly over half of the participants were single, comprising 355 individuals (56.8%), whereas 270 individuals (43.2%) were married. Interestingly, married healthcare workers exhibited more severe depression symptoms compared to their single counterparts in the workplace. Moreover, the participants residing in rented houses faced higher levels of depression, accounting for 27.27%, in contrast to individuals residing in their own houses (26.47%) or those in joint family systems (15.51%).

Significantly higher levels of severe depression were observed among urban healthcare workers (37.71%) compared to those from the rural areas (18.81%). This indicates a noteworthy disparity in depression levels based on the participants' location of residence.

### 3.2. Profession and Work Sector

Our findings revealed that doctors exhibited significantly higher levels of severe depression (33.33%) compared to lab professionals (16.04%) and individuals in other healthcare roles (30.76%). Additionally, health workers employed in the private sector experienced higher levels of depression (38.46%) in comparison to those working in the government sector (15.11%), and this difference was statistically significant. Notably, severe depression levels were found to be more prevalent among health workers in the private sector (38.46%) than among government employees (15.11%). Furthermore, no significant difference in depression levels was observed between professionals with direct patient contact and those without it.

### 3.3. Psychological Factors Associated with Depression among Participants

Among the participants, slightly less than half, 295 individuals (47.2%), contracted COVID-19 infection at their workplace. Among these individuals, 70 (23.72%) reported no depression, while 45 (23.72%) experienced mild depression, 115 (38.98%) experienced moderate depression, and 65 (22.03%) experienced severe depression levels. Additionally, during work, the participants observed that 580 of their colleagues (89.92%) also contracted COVID-19 infection. The relationship between this observation and depression levels in the participants was examined, revealing that 110 individuals (18.96%) reported mild depression, 205 (35.34%) reported moderate depression, and 115 (18.82%) reported severe depression levels (*p*=0.0305).

**Figure 1:**
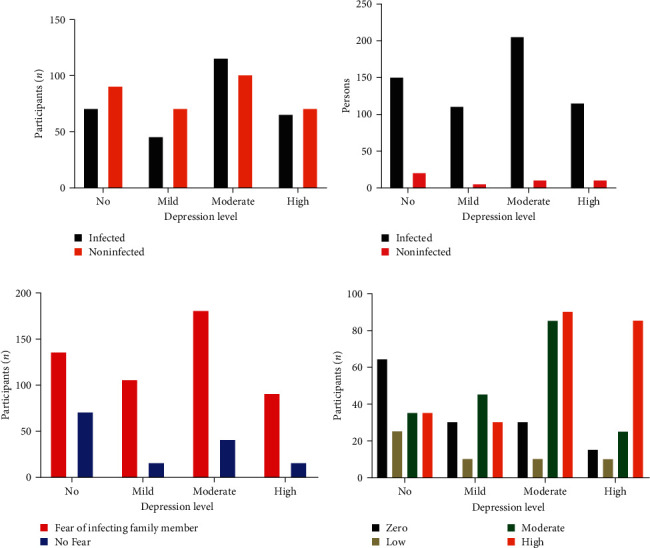
Depression levels among participants attributed to various factors related to COVID-19. (a) Fear of self-infection with COVID-19, (b) comparison of depression levels between participants working with infected colleagues and those working with noninfected colleagues, (c) depression levels among participants fearing the possibility of infecting family members after work, and (d) depression levels among participants arising from the fear of working with COVID-19.

Approximately 80% of the participants expressed fear of infecting their family members due to their work with COVID-19. Among these participants, 25.74% did not experience any symptoms of depression, while 74.75% experienced varying levels of depression (*p*=0.0001). Moreover, more than three-fourths of the participants (77.6%) reported experiencing fear in their workplace. Within this group, 240 individuals (38.4%) reported a high level of fear, while 190 individuals (30.4%) reported a moderate level of fear. It was observed that this fear was associated with depression among the participants, as illustrated in Figure 1.

### 3.4. Working Conditions and Their Relationship to Depression

**Figure 2:**
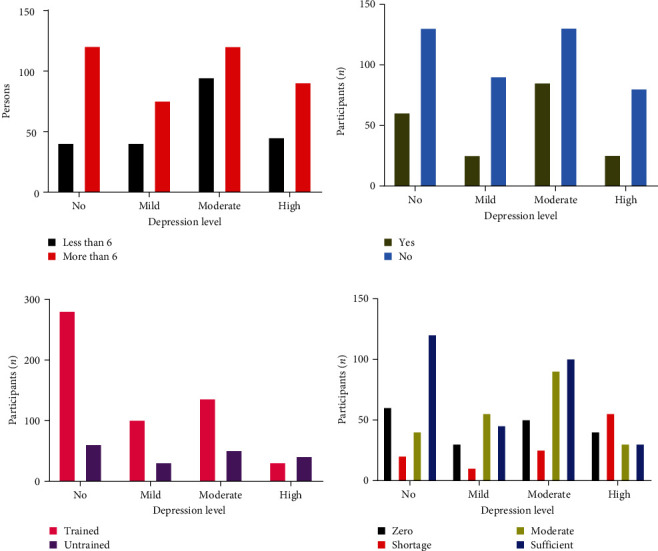
Comparison of workplace parameters affecting depression in workers. (a) Effect of extended working hours on workers' depression levels, (b) impact of negative social support at the workplace on workers' depression, (c) influence of inadequate training and guidance on workers' depression, and (d) relationship between the unavailability of personal protective equipment (PPE) and depression levels among participants.

Figure 2 illustrates the various workplace factors associated with depression among frontline healthcare professionals. Out of the total 625 participants, 405 individuals (64.8%) reported working more than regular hours. Among these individuals, 120 (29.62%) did not experience depression, while 75 (18.51%), 120 (29.62%), and 90 (22.22%) reported mild, moderate, and severe levels of depression during their work with COVID. Notably, prolonged working hours emerged as a significant contributing factor to depression levels in the participants (*p*=0.0017). Ensuring the availability of personal protective equipment, providing adequate training, and implementing measures to reduce extended working hours, organizational support, effective communication, and initiatives fostering cooperation among healthcare teams also play pivotal roles. By addressing these multifaceted factors, a comprehensive approach can contribute significantly in reducing depression levels among healthcare professionals. When it came to receiving social support while working with COVID-19, two-thirds of the participants, 430 individuals (66.66%), reported not receiving any social support. Among these individuals, 130 (30.23%) experienced moderate depression, while 80 (18.60%) experienced severe depression (*p*=0.00024). It was observed that being deprived of social support had a detrimental impact on participants' depression levels. Regarding training to deal with COVID-19, it was found that 440 participants (68.21%) had received training, which helped to reduce their depression levels. Among these trained individuals, 280 (63.63%) reported no depression, and only 30 (6.81%) experienced severe levels of depression. This suggests that training instilled confidence among participants and contributed to a decrease in their depression levels (*p*  < 0.0001). Trainings on the range of topics, including updated protocols for managing COVID-19 cases, proper utilization of personal protective equipment (PPE), infection control measures, and strategies for coping with the psychological stress associated with the pandemic are very necessary for healthcare professionals. The training will equip healthcare professionals with the necessary knowledge and skills to navigate the unique challenges posed by the pandemics.

Exploring the availability of (PPE for frontline healthcare professionals in the workplace and its association with depression levels, it was observed that due to a shortage of PPE, severe depression levels were found in 55 individuals (50%) out of 110 participants (17.05%). Conversely, when high availability of PPE was reported among 290 health workers (44.96%), only 30 individuals (10.34%) experienced severe depression. This association highlighted the significance of adequate PPE availability in mitigating severe depression levels among participants (*p*  < 0.0001).

### 3.5. Association of Social Problems at Work with Mental Health and Professional Work Efficacy of Health Workers

**Figure 3:**
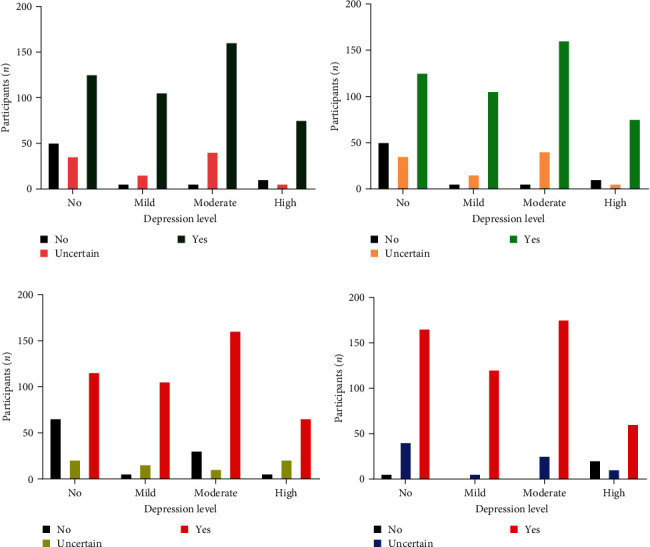
Factors influencing depression and its consequences. (a) Relationship between social problems and depression, (b) correlation between negative social support and depression among frontline healthcare professionals, (c) impact of increased depression levels on physical and psychological health, and (d) effect of depression on reducing professional efficacy in the workplace.

The analysis conducted focused on examining the association between social problems and negative support in the workplace with depressive symptoms, as depicted in Figure 3. Unfortunately, during the pandemic, we observed the presence of social problems at work, which emerged as a primary source of depression. Among the 625 participants, 290 individuals (46.40%) confirmed facing social issues at work, while 130 (20.80%) were uncertain about the question, and 205 (32.80%) did not report experiencing social issues. The analysis revealed a significant association between social problems at work and depression levels among health workers (*p*=0.0001).

Overall, 465 individuals (74.4%) reported facing negative social support in the workplace during the pandemic. Among these individuals, 125 (26.88%) reported no depression, while 105 (22.58%) and 160 (34.40%) experienced mild and moderate levels of depression at work, respectively. This finding highlighted a significant correlation between negative social support and depression levels (*p*  < 0.0001).

Furthermore, we analyzed the physical and physiological health conditions of the workers. Among the 445 health workers (71.2%) who reported facing health problems during the pandemic, 105 (16.8%) did not experience any symptoms. The presence of depressive symptoms in health workers had a negative impact on their physical and physiological health condition (*p*  < 0.0001).

In last, the relationship between depressive symptoms and the professional work efficacy of health workers was assessed. Out of the 520 participants, 432 individuals (83.20%) confirmed a reduction in their work efficacy during the pandemic, while only 25 individuals (4%) responded negatively. Among the 520 participants, 165 (31.73%) reported no depressive symptoms but experienced a decline in their work efficacy during the COVID-19 pandemic. The remaining 520 participants, 125 (20%), 200 (32%), and (14.4%), experienced mild, moderate, and severe depression, respectively, significantly impacting their work efficacy (*p*  < 0.0001).

## 4. Discussion

This study focuses on stress, anxiety, and related factors among healthcare workers in Pakistan during the COVID-19 pandemic. The emergence of infections and illnesses among healthcare workers is a concerning issue. Frontline healthcare workers, who are directly involved in examining, diagnosing, and treating infected patients, are particularly vulnerable. In the absence of preventive measures, standard operating procedures, and proper hand hygiene practices, these frontline healthcare workers face a significant risk of infection. Moreover, they may unknowingly transmit the virus to their families, colleagues, community members, and even other patients in the hospital who are being treated for unrelated conditions.

Just like our study, various research studies conducted in different countries have highlighted the occurrence of anxiety and depression among healthcare workers [8, 30–32]. For instance, a study conducted in China revealed that a significant proportion of healthcare workers experienced symptoms of anxiety and depression, with anxiety affecting 44.6% and depression affecting 50.4% of the participants [8]. Our study also found that frontline healthcare workers are at a greater risk of infection compared to the general community, as they have direct and frequent interactions with patients [33]. Another longitudinal study conducted in China, focusing on patients with confirmed COVID-19, reported that 40 healthcare workers (29%) contracted the novel COVID-19 pneumonia in a hospital-related transmission [34]. In Pakistan, 58 healthcare workers, including 42 doctors, have succumbed to COVID-19, and more than 5,000 healthcare workers have tested positive for the virus as the infection rate continues to rise, the prevalence of anxiety and depression among healthcare workers also increases. Additionally, a study involving 52,730 participants during an epidemic showed a higher risk of psychological distress among female participants compared to males [35]. This finding aligns with published data during epidemics, which suggests that females, including female healthcare workers, are more susceptible to the development of anxiety and depression [8, 10]. The higher levels of anxiety among female healthcare workers, as noted in the study, may stem from a combination of societal expectations, workplace dynamics, and gender-specific challenges. Societal norms and traditional gender roles often place additional caregiving responsibilities on women, contributing to the double burden of managing both work and home. Female healthcare workers may face unique challenges within the healthcare setup. The demands of balancing work and personal life, particularly during a pandemic, can lead to increased stress.

According to the findings of our study, it was observed that 9.6% of healthcare workers had normal anxiety levels, 18.4% experienced mild anxiety, 34.4% reported moderate anxiety, and a significant 40% suffered from severe anxiety during the COVID-19 pandemic. These results align with international studies reporting the incidence of general anxiety disorder (GAD) among healthcare workers ranging from 1.8% to 5.1% [36]. It is noteworthy that increased anxiety symptoms were observed even in healthcare workers without preexisting psychiatric conditions [37].

Our study results indicate a substantial increase in work-related stress among healthcare workers during the COVID-19 pandemic. Comparisons between the earlier SARS outbreak and the current COVID-19 pandemic suggest a significant variation in stress levels, with percentages ranging from 13% to 80%. This finding highlights the elevated levels of anxiety experienced by healthcare workers in Pakistan, despite the relatively low number of COVID-19 cases [38–40].

Current literature consistently indicates that female healthcare workers tend to experience higher levels of anxiety compared to their male counterparts. Furthermore, anxiety levels have been found to be more prevalent among nursing staff compared to other medical and non-medical staff within the healthcare sector. Interestingly, studies have shown that non-medically trained healthcare workers exhibit a higher prevalence of anxiety compared to their medically trained counterparts [41].

Given that healthcare workers operate in high-stress environments, it is natural for them to display behavioral and emotional responses when faced with extreme stress. To support these individuals, it is crucial to implement stress management techniques, such as counseling and psychotherapy, as swift interventions. Addressing the mental health concerns of healthcare workers is of paramount importance for effective pandemic control and prevention [42]. Utilizing online platforms and electronic media to disseminate medical advice on preventing the transmission of diseases between patients and healthcare workers can help alleviate the pressure experienced by these workers. Such measures contribute to a safer working environment and reduce the burden on medical personnel.

To effectively address the psychological crisis faced by healthcare workers during the COVID-19 pandemic, it is crucial to develop a comprehensive psychological crisis intervention plan. This plan should involve the establishment of a specialized mental health intervention medical team that provides virtual training to raise awareness about the psychological impact of stressful events. This training will guide medical workers in understanding and managing their own psychological well-being. Additionally, a psychological assistance hotline should be implemented to enable healthcare workers to discuss their psychological concerns with trained mental health professionals who can provide support and guidance.

Hospitals should adopt measures such as implementing frequent shift systems, ensuring an adequate supply of food and living essentials, and providing pre-job training to help healthcare workers identify and respond to psychological issues in patients, families, and themselves. Moreover, it is essential to have regular visits from psychological counseling psychologists who can lend a listening ear to medical workers, allowing them to share their experiences and provide much-needed emotional support.

In order to address the secondary mental health problems stemming from the COVID-19 pandemic, an urgent psychological crisis intervention model (PCIM) should be developed and implemented through the use of e-technology. This PCIM should integrate teams of physicians, psychiatrists, psychologists/mental health practitioners, and social workers to deliver early psychological intervention to patients, families, and medical staff. Implementing a diverse range of measures across various healthcare settings will facilitate swift and safe early screening, intervention, and subsequent rehabilitation.

Furthermore, it is important to gather epidemiological data on the mental health consequences, psychological impact, psychiatric morbidity, and psychosocial issues related to COVID-19. This data will help to inform the development of screening, assessment, control, treatment plans, management strategies, progress reports, health status updates, prevention measures, and interventions needed to effectively respond to these challenges. The publication of this study serves as an initial step toward providing guidance on addressing the multifaceted mental health dynamics and implementing psychological interventions for medical workers in Pakistan.

## 5. Conclusion

In conclusion, this study examines the factors associated with depression and mental health among healthcare workers during the COVID-19 pandemic and their impact on work efficacy. Our findings highlight that psychological factors, working environment, and social issues significantly influence the mental health of frontline workers. Moreover, social issues and the fear of infection exposure also contribute to a decline in employees' work efficiency. To validate these results, further studies with large sample sizes and follow-ups are necessary.

In light of these findings, it is crucial for the government to implement immediate psychological interventions to promote the mental well-being of healthcare workers in Pakistan. These interventions should address the specific challenges faced by frontline workers and provide necessary support and resources. By prioritizing the mental health of healthcare professionals, we can help mitigate the negative effects of the pandemic on their overall well-being and work performance.

In summary, our study underscores the importance of recognizing and addressing the psychological and social factors affecting the mental health and work efficacy of healthcare workers during the COVID-19 pandemic. Taking proactive measures to support these individuals are essential for maintaining a resilient healthcare system and ensuring the well-being of both the healthcare workforce and the patients they serve.

## Figures and Tables

**Table 1:** Sociodemographic characteristics.

Variables	*n* (%)	Depression level, *n* (%)				*Chi*-square (*X*^2^) df	*p* Value
		No	Mild	Moderate	Severe		
Gender							
Male	445 (71.2%)	120 (26.96%)	85 (19.10%)	155 (34.83%)	85 (19.10%)	**1.615,1**	**0.2038**
Female	180 (28.8%)	45 (25%)	30 (16.6%)	60 (33.33%)	45 (25%)		
Marital status							
Single	355 (56.8%)	105 (29.57%)	45 (12.67%)	85 (23.94%)	35 (9.85%)	**51.16,3**	**<0.0001**
Married	270 (43.2%)	55 (20.37%)	70 (25.92%)	130 (48.14%)	100 (37.03%)		
Housing status							
Own	170 (27.2%)	40 (23.52%)	30 (17.64%)	55 (32.35%)	45 (26.47%)	**41.22,6**	**<0.0001**
Rent	165 (26.4%)	20 (12.12%)	45 (27.27%)	55 (33.33%)	45 (27.27%)		
Join family	290 (46.4%)	100 (34.48%)	40 (13.79%)	105 (36.20%)	45 (15.51%)		
Residential area							
Urban	555 (88.8%)	155 (27.92%)	105 (18.91%)	185 (33.33%)	110 (18.81%)	**20.12,3**	**0.0002**
Rural	70 (11.2%)	5 (7.14%)	10 (14.28%)	30 (42.85%)	25 (35.71%)		
Occupation							
Lab professional	405 (64.8%)	130 (32.09%)	60 (14.81%)	150 (37.03%)	65 (16.04%)	**53.32,6**	**<0.0001**
Doctor	90 (14%)	20 (22.22%)	20 (22.22%)	20 (22.22%)	30 (33.33%)		
Other	130 (20.8%)	10 (7.69%)	35 (26.92%)	45 (34.61%)	40 (30.76%)		
Work sector							
Government	430 (68.8%)	125 (29.06%)	85 (19.76%)	155 (36.04%)	65 (15.11%)	**45.26,3**	**<0.0001**
Private	195 (31.2%)	30 (15.38%)	30 (15.38%)	60 (30.76%)	75 (38.46%)		
Job status							
Permanent	180 (28.8%)	50 (27.77%)	15 (8.33%)	80 (44.44%)	35 (19.44%)	**22.35,3**	**<0.0001**
Temporary	445 (71.2%)	110 (24.71%)	100 (22.47%)	135 (30.33%)	100 (24.71%)		
COVID contact							
Direct contact	500 (80%)	145 (29%)	95 (19%)	180 (36%)	80 (16%)	**3.895,3**	**0.273**
No contact	125 (20%)	45 (36%)	20 (16%)	35 (28%)	20 (16%)		

Statistically significant differences: *p* Value < 0.05.

## Data Availability

The raw data used to support the findings of this study are available from the corresponding author upon request

## Additional Points

*Limitations of the Study*. The present study has some limitations that should be acknowledged. First, the sampling approach used in this study limits its generalizability to a broader population. The study design is exploratory in nature, aiming to provide insights into the mental health status of healthcare workers. Additionally, being an online study with a self-administered questionnaire, there is a possibility of biased responses. Conducting the study offline was challenging due to the unavailability of participants, which further contributed to the online approach. The study lacked information regarding the specific nature of healthcare workers' jobs and their pre-existing mental health status. Despite these limitations, the findings of this study can serve as a catalyst for governmental action in implementing psychological interventions to alleviate mental health problems among healthcare workers.

## References

[1] Wang C., Horby P. W., Hayden F. G., Gao G. F. (2020). A novel coronavirus outbreak of global health concern. *The Lancet*.

[2] Mahase E. (2020). China coronavirus: WHO declares international emergency as death toll exceeds 200. *British Medical Journal*.

[3] Chong B. S. W., Tran T., Druce J., Ballard S. A., Simpson J. A., Catton M. (2020). Sample pooling is a viable strategy for SARS-CoV-2 detection in low-prevalence settings. *Pathology*.

[4] Zheng Y., Xu H., Yang M. (2020). Epidemiological characteristics and clinical features of 32 critical and 67 noncritical cases of COVID-19 in Chengdu. *Journal of Clinical Virology*.

[5] Que J., Shi L., Deng J. (2020). Psychological impact of the COVID-19 pandemic on healthcare workers: a cross-sectional study in China. *General Psychiatry*.

[6] Shaukat N., Ali D. M., Razzak J. (2020). Physical and mental health impacts of COVID-19 on healthcare workers: a scoping review. *International Journal of Emergency Medicine*.

[7] Liu X., Kakade M., Fuller C. J. (2012). Depression after exposure to stressful events: lessons learned from the severe acute respiratory syndrome epidemic. *Comprehensive Psychiatry*.

[8] Lai J., Ma S., Wang Y. (2020). Factors associated with mental health outcomes among health care workers exposed to coronavirus disease 2019. *JAMA Network Open*.

[9] Chong M.-Y., Wang W.-C., Hsieh W.-C. (2004). Psychological impact of severe acute respiratory syndrome on health workers in a tertiary hospital. *British Journal of Psychiatry*.

[10] Maunder R., Hunter J., Vincent L. (2003). The immediate psychological and occupational impact of the 2003 SARS outbreak in a teaching hospital. *Canadian Medical Association Journal*.

[11] Ho K. Y., Singh K. S., Habib A. G. (2004). Mild illness associated with severe acute respiratory syndrome coronavirus infection: lessons from a prospective seroepidemiologic study of health-care workers in a teaching hospital in Singapore. *The Journal of Infectious Diseases*.

[12] Islam M. S., Kamal A.-H. M., Kabir A. (2021). COVID-19 vaccine rumors and conspiracy theories: the need for cognitive inoculation against misinformation to improve vaccine adherence. *PLOS ONE*.

[13] Pfefferbaum B., North C. S. (2020). Mental health and the COVID-19 pandemic. *New England Journal of Medicine*.

[14] Ali A., Sohaib M., Iqbal S., Hayat K., Khan A. U., Rasool M. F. (2021). Evaluation of COVID-19 disease awareness and its relation to mental health, dietary habits, and physical activity: a cross-sectional study from Pakistan. *The American Journal of Tropical Medicine and Hygiene*.

[15] Maunder R. (2004). The experience of the 2003 SARS outbreak as a traumatic stress among frontline healthcare workers in Toronto: lessons learned. *Philosophical Transactions of the Royal Society of London. Series B: Biological Sciences*.

[16] Stephan U., Zbierowski P., Pérez-Luño A. (2022). Act or wait-and-see ? Adversity, agility, and entrepreneur wellbeing across countries during the COVID-19 pandemic. *Entrepreneurship Theory and Practice*.

[17] Wu P., Fang Y., Guan Z. (2009). The psychological impact of the SARS epidemic on hospital employees in China: exposure, risk perception, and altruistic acceptance of risk. *The Canadian Journal of Psychiatry*.

[18] Shigemura J., Ursano R. J., Morganstein J. C., Kurosawa M., Benedek D. M. (2020). Public responses to the new coronavirus 2019 (2019-nCoV) in Japan: consequences for mental health and target populations. *Psychiatry and Clinical Neurosciences*.

[19] Esterwood E., Saeed S. A. (2020). Past epidemics, natural disasters, COVID19, and mental health: learning from history as we deal with the present and prepare for the future. *Psychiatric Quarterly*.

[20] Magnavita N., Soave P. M., Antonelli M. (2022). Treating anti-vax patients, a new occupational stressor—data from the 4th wave of the prospective study of intensivists and COVID-19 (PSIC). *International Journal of Environmental Research and Public Health*.

[21] Jamal S. (2020). COVID-19: 58 medical workers die fighting coronavirus in Pakistan.

[22] Lung F.-W., Lu Y.-C., Chang Y.-Y., Shu B.-C. (2009). Mental symptoms in different health professionals during the SARS attack: a follow-up study. *Psychiatric Quarterly*.

[23] Maurer D. M., Raymond T. J., Davis B. N. (2018). Depression: screening and diagnosis. *American Family Physician*.

[24] Levis B., Benedetti A., Thombs B. D. (2019). Accuracy of patient health questionnaire-9 (PHQ-9) for screening to detect major depression: individual participant data meta-analysis. *British Medical Journal*.

[25] Sawaya H., Atoui M., Hamadeh A., Zeinoun P., Nahas Z. (2016). Adaptation and initial validation of the patient health questionnaire-9 (PHQ-9) and the generalized anxiety disorder-7 Questionnaire (GAD-7) in an Arabic speaking Lebanese psychiatric outpatient sample. *Psychiatry Research*.

[26] Gallis J. A., Maselko J., O’Donnell K. (2018). Criterion-related validity and reliability of the Urdu version of the patient health questionnaire in a sample of community-based pregnant women in Pakistan. *PeerJ*.

[27] Ghafoor H., Ahmad R. A., Nordbeck P., Ritter O., Pauli P., Schulz S. M. (2019). A cross-cultural comparison of the roles of emotional intelligence, metacognition, and negative coping for health-related quality of life in German versus Pakistani patients with chronic heart failure. *British Journal of Health Psychology*.

[28] Lwanga S. K., Lemeshow S. (1991). *Sample Size Determination in Health sStudies: A Practical Manual*.

[29] Naser A. Y., Dahmash E. Z., Al-Rousan R. (2020). Mental health status of the general population, healthcare professionals, and university students during 2019 coronavirus disease outbreak in Jordan: a cross-sectional study. *Brain and Behavior*.

[30] Pappa S., Ntella V., Giannakas T., Giannakoulis V. G., Papoutsi E., Katsaounou P. (2020). Prevalence of depression, anxiety, and insomnia among healthcare workers during the COVID-19 pandemic: a systematic review and meta-analysis. *Brain, Behavior, and Immunity*.

[31] Hu D., Kong Y., Li W. (2020). Frontline nurses’ burnout, anxiety, depression, and fear statuses and their associated factors during the COVID-19 outbreak in Wuhan, China: a large-scale cross-sectional study. *eClinicalMedicine*.

[32] Gupta A. K., Mehra A., Niraula A. (2020). Prevalence of anxiety and depression among the healthcare workers in Nepal during the COVID-19 pandemic. *Asian Journal of Psychiatry*.

[33] Nguyen L. H., Drew D. A., Graham M. S. (2020). Risk of COVID-19 among front-line health-care workers and the general community: a prospective cohort study. *The Lancet Public Health*.

[34] Bhatti M. (2020). Pakistan has lost 42 doctors among 58 healthcare providers to COVID-19.

[35] Qiu J., Shen B., Zhao M., Wang Z., Xie B., Xu Y. (2020). A nationwide survey of psychological distress among Chinese people in the COVID-19 epidemic: implications and policy recommendations. *General Psychiatry*.

[36] Pirkola S. P., Isometsä E., Suvisaari J. (2005). DSM-IV mood-, anxiety- and alcohol use disorders and their comorbidity in the Finnish general population. *Social Psychiatry and Psychiatric Epidemiology*.

[37] Ho C. S., Chee C. Y., Ho R. C. (2020). Mental health strategies to combat the psychological impact of coronavirus disease 2019 (COVID-19) beyond paranoia and panic.. *Annals of the Academy of Medicine, Singapore*.

[38] Maunder R. G. (2009). Was SARS a mental health catastrophe ?. *General Hospital Psychiatry*.

[39] Chan A. O. M., Huak C. Y. (2004). Psychological impact of the 2003 severe acute respiratory syndrome outbreak on health care workers in a medium size regional general hospital in Singapore. *Occupational Medicine*.

[40] Phua D., Tang H., Tham K. (2005). Coping responses of emergency physicians and nurses to the 2003 severe acute respiratory syndrome outbreak. *Academic Emergency Medicine*.

[41] Wang W., Song W., Xia Z. (2020). Sleep disturbance and psychological profiles of medical staff and non-medical staff during the early outbreak of COVID-19 in Hubei Province, China. *Frontiers in Psychiatry*.

[42] Kontoangelos K., Economou M., Papageorgiou C. (2020). Mental health effects of COVID-19 pandemia: a review of clinical and psychological traits. *Psychiatry Investigation*.

